# New ways of thinking for sustainable oral health: how integrating oral, respiratory, and postural health into early childhood development can drive innovation and research

**DOI:** 10.3389/fdmed.2025.1659546

**Published:** 2025-09-15

**Authors:** Valentina Gecha, Roger Price

**Affiliations:** Non-profit International Institute for OrthoPostural Education Ltd., Sydney, NSW, Australia

**Keywords:** breathing, breathing disordered sleep, early childhood development and education, functional health screening, oral health, orthopostural education, planetary health, posture

## Abstract

This Perspective presents an emerging integrative model for early childhood health promotion, developed within For the Health of Future Generations (HFG) initiative. The model translates evolving definitions of oral health into culturally adaptive, school-based interventions that integrate oral, respiratory, postural, and musculoskeletal health within early learning systems. Rooted in transdisciplinary collaboration, the HFG protocol promotes upstream prevention through functional health routines, health literacy, and early observation. Preliminary field experience from the ongoing four-year, multi-country pilot program in diverse early learning settings revealed concerning patterns in children's functional health and behavioral readiness for school, as perceived by educators and caregivers. Interviews suggest increasing challenges in school transition and a lack of physiological maturity among children. These findings underscore the need for integrative, developmentally supportive strategies in early education. By embedding health-enabling practices into early learning environments, the HFG model offers a pathway toward more equitable, sustainable child development frameworks aligned with WHO priorities and the United Nations Sustainable Development Goals (SDGs) 3, 4, 11, and 13.

## 1 Introduction. New paradigms in oral health

Although the definitions of oral health proposed by the World Health Organization (WHO) and the FDI World Dental Federation (FDI) are broadly similar, they differ in one important respect. This distinction has implications for how oral health is integrated into programmes and interventions. According to the WHO, oral health is “the state of the mouth, teeth and orofacial structures that enables individuals to perform essential functions, such as eating, breathing and speaking” (1). The FDI, by contrast, highlights oral health as multifaceted and linked to a wider set of abilities. Both organisations acknowledge the role of environmental and social determinants (2), but the WHO definition goes further by explicitly including breathing and musculoskeletal structures, positioning oral health as part of systemic health. These determinants are increasingly shaped by global industrialisation (3, 4), which alters environments, dietary patterns, and physical activity, while increasing exposure to processed foods (5). Such dynamics highlight the relevance of Planetary Health principles, which emphasise the interdependence between human health, natural systems, and environmental change.

This Perspective introduces an integrative model for early childhood health promotion, developed within the Health for the Future Generations (HFG) initiative. The model responds to current gaps in oral health strategies, which often remain siloed and detached from broader child development. It translates evolving definitions of oral health into practice currently piloted within a four-year, multi-country program, with additional sites onboarding over time in early learning settings. These interventions intentionally connect oral, respiratory, postural, and musculoskeletal health within early learning systems, positioning early learning institutions as platforms for upstream prevention. To ensure relevance across contexts, the model applies context-sensitive adaptation, defined as “the systematic modification of an evidence-based treatment (EBT) or intervention protocol to consider language, culture, and context in such a way that it is compatible with the person's cultural patterns, meanings, and values” (6). Across the program period, sites will recruit children from diverse socio-cultural, linguistic, and socioeconomic groups to enable pooled cross-site analyses and equity-focused insights.

## 2 The forgotten physiology of breathing and its developmental impact

Although breathing is essential for health, it is often overlooked in pediatric, dental, and educational fields. Nasal breathing is the physiological norm in mammals, providing effective conditioning of inspired air. The nasal cavity, with its specialized anatomical and mucosal structures, plays a critical role in warming, humidifying, and filtering inhaled air before it reaches the lungs. As described in Gray's Anatomy, the vibrissae (nasal hairs) and mucosal secretions help trap and neutralize airborne particles and pathogens; the turbinates increase the surface area for air contact, facilitating temperature adjustment and humidification; and the paranasal sinuses contribute to moistening inhaled air (7). The tonsils, as part of the lymphoid tissue of Waldeyer's ring, form a physical immune barrier against microbes entering the lower respiratory tract, complementing the nasal and sinus defenses described above (8). Together with the lymphoid tissues of the pharynx, these mechanisms form a multi-layered barrier that protects the respiratory tract from contaminants and infection. In addition, epithelial cells in the nasal passages release nitric oxide (NO), a gaseous mediator with antimicrobial and vasodilatory effects, which supports host defense in the upper airway (9).

Mouth breathing bypasses this process. Unfiltered air dries the oral cavity, alters saliva's pH, and suppresses its antimicrobial properties. These effects contribute to microbial dysbiosis, decay, halitosis, and upper airway inflammation (10–12). Nocturnal mouth breathing disrupts salivary regulation and triggers unnecessary nighttime water intake. Combined with sympathetic nervous system activation and potential vasoconstriction of the bladder (13), this can contribute to bed wetting. Importantly, in children with habitual snoring and mouth breathing were both significantly associated with nocturnal enuresis (14).

Furthermore, oral (mouth) breathing disrupts the balance of carbon dioxide—central to the Bohr Effect, the mechanism that facilitates oxygen release from hemoglobin to body tissues (13). When CO₂ levels fall, hemoglobin retains oxygen more tightly, compromising delivery to vital organs, especially the brain. Even mild chronic reductions in oxygen availability during childhood can impair neural development. This may result in measurable deficits in learning, attention, emotional self-regulation, and behavioral control. Persistent mouth breathing has been strongly associated with breathing-related sleep disruption (commonly classified under “sleep disordered breathing”), which further exacerbates cognitive challenges, mood instability, reduced learning capacity, and susceptibility to recurrent respiratory infections (15, 16).

Air pollution exacerbates these problems. Children exposed to polluted environments are more likely to develop upper respiratory tract infections (URTIs), especially when coupled with dysfunctional breathing patterns (17–20). The intersection of environmental pollution and poor functional breathing highlights the urgent need for prevention strategies rooted in both health promotion and environmental sustainability.

This reinforces the relevance of the HFG model within the framework of Planetary Health and WHO's 2022 report on «Climate Change and Noncommunicable Diseases (NCDs)». These insights call for a reconfiguration of how oral health is taught, assessed, and promoted in childhood. The integration of breathing physiology into oral health education—especially within early learning environments—offers a critical opportunity to address upstream risks before dysfunction becomes entrenched. The HFG model responds to this gap by embedding respiratory awareness and functional breathing into daily routines, empowering educators as proactive health enablers.

## 3 Lymphatic flow, diaphragmatic function, and postural feedback loops

Breathing is intrinsically linked to immune function via lymphatic drainage. The diaphragm functions as a primary mechanical pump for lymphatic circulation, an effect particularly important for lymphatic clearance from the thoracic and abdominal regions (21). Nasal-diaphragmatic breathing, by encouraging efficient diaphragmatic excursion, is thought to optimize lymph flow and the removal of metabolic by-products, further supporting systemic immune regulation (13). Chronic mouth breathing disrupts this pattern, reducing diaphragmatic efficiency and leading to compensatory changes in posture and ventilation. Evidence shows that children who habitually breathe through the mouth often develop forward head posture and altered pulmonary mechanics, which correlate with measurable reductions in lung function parameters such as forced vital capacity (FVC) (22, 23).

These maladaptive patterns may contribute to stagnation of lymphatic flow and create a pro-inflammatory environment in lymphoid tissues, especially the tonsils and adenoids (24, 25). In the absence of nitric oxide (NO) stimulation from nasal airflow, these tissues are more vulnerable to hypertrophy and infection (26). Together, these interrelated dysfunctions illustrate how postural and breathing habits contribute to chronic inflammatory conditions. Addressing such patterns early in life may help prevent cascading physiological stress and reduce future healthcare burdens (27).

These processes can evolve into self-perpetuating cycles: lymphatic congestion worsens respiratory obstruction, which in turn reinforces postural compensation and the overuse of accessory breathing muscles (e.g., sternocleidomastoid, intercostals). This pattern contributes to fatigue, pain, and dysfunctional posture, consistent with previous descriptions of vicious cycles in mouth breathing and craniofacial development (28) and compensatory respiratory muscle recruitment (29).

The HFG protocol responds directly to this challenge by embedding respiratory awareness and posture-focused strategies into early learning settings. Through playful movement, guided breathing, and structured educator-led observation, the program aims to interrupt maladaptive cycles before they consolidate. This positions early childhood education as a frontline setting for systemic health prevention and highlights the need for integrative, cross-disciplinary approaches to health promotion. Within this framework, we introduce the concept of orthopostural education, defined as the structured integration of breathing and posture-oriented routines into early learning, aiming to reinforce functional stability as a foundation for child development.

## 4 Behavioral and developmental consequences of dysfunctional breathing

Children who breathe through the mouth often exhibit hyperactivity, poor concentration, and behavioral issues that mimic ADHD or emotional disorders (30). These signs are often misdiagnosed while the root cause—postural compensations to facilitate breathing, leading to disordered breathing and sleep patterns (22, 23)—go untreated.

Such patterns become neurologically entrenched if not corrected early (16, 17). Unfortunately, current medical and dental training seldom addresses these upstream causes. Otorhinolaryngology remains surgically focused, with adenoidectomy and tonsillectomy as default treatments. Yet, research suggests that approximately 50% of children experience recurrence within 1–2 years post-surgery (31). This underscores the importance of upstream, function-focused prevention in early learning settings, rather than relying solely on surgical interventions. While surgery may be necessary in some cases, it does not by itself address the underlying functional disturbances. A systems-level shift is needed—from symptom treatment to sustained functional rehabilitation, supporting healthy breathing and postural development.

By embedding preventive, function-oriented routines into early learning settings, the HFG protocol addresses not only physiological function but also supports neurodevelopment and school readiness, reducing the risk of long-term behavioral and learning difficulties.

## 5 Implications for prevention and system integration

The impact of dysfunctional breathing extends beyond the oral cavity. Recurrent infections, oral microbiome disruption, and nasopharyngeal inflammation have all been linked to chronic mouth breathing and environmental exposures such as air pollution. In addition, poor posture has been shown to co-occur with dysfunctional breathing patterns in children (32). Prevention begins with function, which in turn begins with breath. Breath, when supported by posture and behavior, lays the foundation for lifelong resilience (5).

By targeting modifiable behaviors through culturally sensitive, repeatable routines in early learning settings, the HFG model promotes systemic health. Training local medical teams to recognize dysfunctional breathing and related adaptations, while equipping educators to embed functional routines—such as guided breathing, posture games, and daily oral hygiene—enhances children's learning capacity and contributes to the long-term prevention of noncommunicable diseases (NCDs). Furthermore, the model operationalizes early prevention strategies consistent with Planetary Health principles and SDG 3, emphasizing that child health is inseparably linked to environmental stability.

## 6 Framing oral health through a transdisciplinary lens

Conventional oral health strategies have largely focused on detecting and treating disease once clinical signs appear, typically within dental settings. In contrast, the HFG protocol advances a preventive, health-oriented model that prioritizes early functional development. It introduces upstream interventions that strengthen core physiological functions—such as breathing, posture, and chewing—during the critical early years, before dysfunction or pathology emerges.

This transdisciplinary approach builds on evolving definitions of oral health proposed by WHO and the FDI. Both define oral health not only as the absence of disease, but as a dynamic state encompassing physical, psychological, and functional well-being, including the ability to breathe, chew, speak, and participate fully in society. These definitions position oral health as a core component of overall health and align with Strategic Objective 3 of the WHO Global Oral Health Strategy, which emphasizes cross-sector engagement beyond dentistry.

By translating these definitions into practice within early learning settings, the HFG protocol engages oral health professionals, educators, caregivers, allied health specialists (e.g., speech therapists, physiotherapists, nutritionists), and policy-makers. This reflects a shift toward integrated, person-centered care aligned with the principles of transdisciplinary collaboration, as highlighted in recent work by WHO and the U.S. National Academies of Sciences, Engineering, and Medicine (NASEM) on interprofessional collaboration (33, 34). The protocol transforms abstract definitions into culturally relevant routines, enabling early learning settings to serve as platforms for early intervention and equity in child development.

The model also aligns with WHO's Nurturing Care Framework (27), which underscores the importance of early intervention, responsive caregiving, and safe, stimulating environments. It further aligns with the SDGs, particularly SDG 3 (health), SDG 4 (education), SDG 10 (reduced inequalities), SDG 11 (sustainable communities), and SDG 13 (climate action). The HFG model contributes to these goals through the integration of oral and functional health promotion into education systems, the reduction of long-term health disparities, and the use of inclusive, low-resource methods that can be embedded in community structures (Table 1).

**Table 1:** Alignment with specific SDGs.

SDG	Focus	How the HFG Protocol addresses it
3	Good Health and Well−being	Promotes functional health foundations in early childhood (breathing, posture, oral health)
4	Quality Education	Integrates health literacy into daily routines and early learning curricula
10	Reduced Inequalities	Targets health promotion in underserved early learning populations through culturally adapted protocols
11	Sustainable Cities and Communities	Engages local institutions (municipalities, early learning institutions) in co−delivery of child health promotion
13	Climate Action	Utilizes low−resource, non−invasive interventions compatible with eco−friendly implementation

Building on this foundation, the next section outlines how the HFG protocol has been translated into practice. Its multi-region implementation supports iterative refinement across diverse early learning systems and income settings, laying the groundwork for sustained evaluation and staged scale-up throughout the program.

## 7 Concept to practice

HFG protocol, built on the principles of orthopostural education, was developed in response to the need for a comprehensive, culturally adaptable framework linking early childhood education with functional health promotion. Its design draws on the FDI definition of oral health, the WHO Global Oral Health Strategy, and recommendations from the National Academies of Sciences, Engineering, and Medicine (NASEM) reports. A multidisciplinary team of experts in pediatrics, dentistry, education, and public health co-designed the model, which is currently being piloted across multiple regions in a staged, multi-country rollout with additional countries continuing to onboard over the four-year program; each locale contributes to iterative refinement at different stages of the program. Field observations, early feedback from educators and families, and stakeholder consultations continue to shape its structure and rollout.

The protocol targets children in early learning (3–7 years) and is structured around three standardized components:

1. **Daily health routines**—guided breathing exercises (before/after rest), posture and movement games, supervised toothbrushing, and chewing activities to reinforce functional habits through play.
2. **Health literacy for families and educators**—illustrated stories, songs, classroom posters, caregiver habit trackers and take-home activities, and teacher workshops that integrate routines into pedagogy.
3. **Functional assessments**—semi-annual observation cycles conducted through five standardized HFG stations: S1 Baseline Measurements, S2 Oral and Dental Health Assessment, S3 Posture Analysis, S4 Capnometry, and S5 General Functional Assessment. These assessments use validated indices (dmft, VPI), developmental and behavioral scales (e.g., DECA), and structured motor coordination tasks, supported by observational checklists for breathing and posture.

Harmonized protocols and shared training enable comparable implementation, while site-specific adaptation ensures relevance; over the four-year period, recruitment aims to achieve representation across diverse population groups and contexts. Implementation to date has engaged over 200 children across multiple early learning settings (state and private), supported by teams including pediatricians, dentists, orthodontists, physiotherapists, speech and language therapists, and public health specialists. Educators receive tailored methodological guidelines, and parents are involved through orientation sessions. All components are intentionally designed to be culturally adaptable, low-cost, and replicable, ensuring feasibility in diverse socioeconomic contexts.

## 8 Implementation case study: Romania

Early engagement in one pilot region served as an illustrative case study for how functional health promotion can be positioned within broader child development policies (1, 2). Rather than treating oral health or any single function as an isolated intervention, the HFG protocol was framed as part of a wider agenda addressing school readiness, developmental equity, and systemic prevention of non-communicable diseases. This holistic positioning allowed the program to enter local policy dialogues not only in education but also in public health and child development.

Stakeholder engagement was guided by principles of sustainable health promotion—emphasizing capacity-building, shared ownership, and long-term relevance. The Romanian pilot, one of several regional entry points, served as an illustrative case study, highlighted the potential for educators to act as frontline enablers of functional health, integrating daily routines that support posture, breathing, oral function, and motor coordination. Their engagement illustrates how strengthening capacity in early learning settings can extend the reach of preventive strategies far beyond the clinical sphere. Parents were introduced to the program through dedicated onboarding and culturally adapted materials, while local field experts contextualized the functional assessments for early learning environments. Lessons learned here inform the broader multi-country program alongside parallel work in other regions.

## 9 Early experiences from pilot implementation

Early experiences across pilot sites—spanning multiple regions, with one offering the most developed case to date—reveal practical entry points for embedding functional health practices in public education. It also suggests that cross-sectoral alignment—especially at the municipal level—may be critical for long-term adoption and impact. It illustrates the potential for early learning institutions to become platforms for delivering functional health promotion.

The following table summarizes initial results and mutual gains across key stakeholder groups involved in the Romanian pilot implementation (Table 2). The project's contribution extends beyond the individual child. By building functional health literacy among caregivers and educators, the model addresses upstream determinants of health inequities. Children who develop core functional skills early are consistently shown to be better positioned to succeed academically, socially, and emotionally—early childhood, up to 8 years, is a critical period for cognitive, social, emotional and physical development (35, 36).

**Table 2:** Stakeholder engagement, contributions, and outcomes in the implementation of the HFG protocol.

Stakeholder	Role and support in implementation	Initial results/observed outcomes	Long−term benefits/co−benefits
Municipal Authorities	Enabled access to early learning institutions; framed within policy	Protocol integrated into early learning policy	Strengthened local leadership in health promotion
Educators	Delivered daily routines; supported data collection; received training; liaised with families	Developed new competencies; embedded routines in classrooms	Recognition as health facilitators
Parents/Caregivers	Participated in screenings; received communication materials	Engaged with home–school continuity	Improved understanding of child health needs
Field Experts	Adapted tools to local context; coordinated assessments	Ensured cultural relevance and clinical validity	Sustained evidence−based practice
Children	Engaged in games and structured routines	Improved posture, breathing, hygiene, and school readiness	Early adoption of functional habits

This approach illustrates the potential to advance a whole-health paradigm by positioning early learning as a platform for systemic health equity and resilience, aligning with international frameworks such as UNICEF's Early Childhood Development agenda (37) and the WHO Nurturing Care Framework (38).

## 10 Conclusion

This Perspective outlines a transdisciplinary approach to translating the evolving definition of oral health into early learning practice. By intentionally embedding oral, respiratory, postural, and musculoskeletal health into early learning environments, the HFG model operationalizes early prevention and offers a feasible, equity-oriented pathway to strengthen developmental resilience from the earliest years. Future priorities should include cultural and policy adaptation, longitudinal evaluation of outcomes, and systematic integration into educator preparation and health professional training. Effective implementation will depend on coordinated action across health and education sectors, supported by alignment with planetary health priorities. As a four-year, multi-country program designed for ongoing expansion across diverse regions, the initiative reflects a planetary health perspective.

By aligning oral health with systemic physiological functions and upstream determinants—including environmental and climate-related factors—the HFG approach illustrates the potential to advance a whole-health paradigm. It shifts the focus from treating dysfunction to enabling resilience, positioning early learning as a strategic platform for sustainable and equitable child health promotion.

## Data Availability

The raw data supporting the conclusions of this article will be made available by the authors, without undue reservation.
